# Impact of Demographic, Clinical, and Preventive Factors on Caries Susceptibility and Cavitation in a Six-to-15-Year Cohort

**DOI:** 10.7759/cureus.72917

**Published:** 2024-11-03

**Authors:** Prabhat Kumar, Gaurav Verma, Pranamee Barua, Satya P Singh, Afshan Khan, Parveen Naja SK, Seema Gupta

**Affiliations:** 1 Department of Pedodontics and Preventive Dentistry, Dental College Azamgarh, Azamgarh, IND; 2 Department of Conservative Dentistry and Endodontics, Mithila Minority Dental College and Hospital, Darbhanga, IND; 3 Department of Pedodontics and Preventive Dentistry, Regional Dental College, Guwahati, IND; 4 Department of Orthodontics, Kothiwal Dental College and Research Centre, Moradabad, IND

**Keywords:** assessment, caries, children, risk, susceptibility

## Abstract

Introduction: Dental caries continues to represent one of the most widespread oral health challenges on a global scale, particularly in adolescents. This cross-sectional investigation examines the impact of demographic, clinical, and preventive factors on caries susceptibility and cavitation in children aged six to 15 years, thereby offering a thorough analysis of risk patterns and preventive strategies.

Materials and methods: This prospective, cross-sectional study was conducted on 2,000 schoolchildren, aged six to 15 years. Demographic and clinical data and data on preventive factors were gathered using the American Academy of Pediatric Dentistry (AAPD) Caries-Risk Assessment Tool (CAT). Based on clinical assessments and self-reported behaviors, participants were classified into high-, medium-, and low-risk groups. Statistical methodologies, including chi-square analyses and odds ratios (OR), were employed to evaluate the significance of the relationships among the variables.

Results: The associations between gender, educational attainment, and caries risk were statistically significant (p = 0.001), demonstrating that females exhibited a greater vulnerability than their male counterparts. Individuals enrolled in higher education levels showed an increased prevalence of high-risk categorization. Notable variations among communities were also observed, with specific demographics presenting a heightened susceptibility to caries. The frequency of tooth brushing, sugar intake, and regular dental examinations exhibited strong correlations with caries risk, whereas cavitation was recorded in 65.50% of the subjects. Key predictors included visible plaque, diminished salivary flow, and use of orthodontic appliances (p = 0.001). Preventive strategies, such as the application of fluoridated products and daily tooth brushing, have been found to significantly mitigate the risk of cavitation.

Conclusion: This investigation emphasizes the complex, multifaceted nature of caries susceptibility, accentuating the critical role of demographic, clinical, and preventive factors.

## Introduction

Dental caries, commonly referred to as tooth decay, represents a critical public health concern that affects individuals of diverse age demographics, with a notably heightened prevalence among pediatric and adolescent populations. This condition is characterized as a multifactorial disease, marked by the demineralization of dental enamel, which results from acids produced by oral microbiota, primarily *Streptococcus mutans* and *Lactobacillus* [1]. In the pediatric demographic, if caries remain unaddressed, it can lead to significant adverse outcomes, such as absenteeism from school, suboptimal academic achievement, and diminished self-worth stemming from dental discomfort and aesthetic issues. Among school-aged children, particularly those in the six-to-15-year age range, the likelihood of developing dental caries is frequently exacerbated by physiological, behavioral, and social influences, thus necessitating targeted risk evaluations and preventive interventions [2,3].

Inadequate oral hygiene practices, excessive consumption of sugary foods and drinks, irregular dental examinations, and hormonal changes related to puberty considerably increase vulnerability to dental caries during this critical developmental period. In numerous regions, the prevalence of dental caries among adolescents remains at a distressing high level [2]. Arangannal et al. [3] documented the prevalence of dental caries in children aged six years to be 57%, at seven years, 67%, at eight years, 63%, at nine years, 74%, at 10 years, 76%, at 11 years, 74%, at 12 years, 69%, at 13 years at 71%, and 69% at 14 years.

Caries risk evaluation represents a critical instrument for discerning individuals who are at an elevated risk for the onset of dental caries. This process entails the assessment of an array of biological, environmental, behavioral, and socioeconomic variables that affect the probability of caries development [4]. For the pediatric population aged between six and 15 years, a comprehensive risk evaluation can facilitate the formulation of tailored preventive interventions, which may encompass fluoride treatments, sealant applications, nutritional guidance, and encouragement of proficient oral hygiene practices [5]. Through the early identification of individuals at elevated risk, it is feasible to administer interventions aimed at either preventing the emergence of caries or impeding its advancement, thereby enhancing oral health outcomes and diminishing the necessity for more invasive therapeutic measures in subsequent stages.

This study aimed to evaluate the susceptibility to dental caries among children aged six to 15 years, using a caries risk assessment tool, to study the interrelationship between caries risk indicators, caries risk factors, and overall caries risk, with a concentrated examination of both intrinsic and extrinsic determinants that may influence the onset of carious lesions. The secondary objective was to classify them into different risk groups so that effective public health strategies could be implemented, and to foster equitable access to dental care services.

## Materials and methods

Study design

This cross-sectional study was conducted in the Department of Pedodontics and Preventive Dentistry, Kothiwal Dental College and Research Centre, Moradabad, from January 2022 to December 2023. The study was executed within a randomly chosen cohort of 20 schools, thereby guaranteeing representation from both urban and rural environments. The research methodology was formulated to encompass a comprehensive assessment of oral health, gathering sociodemographic data, and identifying behavioral risk determinants linked to dental caries. Information was collected through clinical evaluations, organized interviews, and surveys. Ethical committee approval was obtained before starting this study (KDCRC/IERB/11/2021/28). Consent from parents/guardians and assent from children were obtained. The confidentiality of participants' data was maintained throughout the study.

Study population

The target population consisted of schoolchildren aged six to 15 years who were enrolled in both public and private schools. The inclusion criteria were age between six and 15 years and enrolment in selected schools. Children with systemic illnesses, developmental anomalies, and those with extensive dental work beyond simple restorations were excluded from the study. These exclusions were set to avoid confounding factors that could distort the caries risk assessment.

Sample size determination

The sample size was calculated using a standard formula for a prospective cohort study, ensuring a confidence level of 95% and a margin of error of 2%. The present study used a prevalence rate of 67% for caries in school-going children aged six to 12 years [6]. After adjustments for possible dropouts and non-responses, approximately 2,000 children were included in the study. Stratified sampling was employed to ensure that an equal number of participants were drawn from both urban and rural areas and from both genders to reflect the diversity of the population.

Methodology and data collection

The evaluation of caries risk encompassed (1) a structured questionnaire, (2) a comprehensive interview, (3) an assessment of oral hygiene practices, (4) a clinical examination, and (5) the formulation of a risk profile for each child. A structured questionnaire was administered to gather data on the various risk factors for dental caries. The questionnaire was completed by the parents/guardians of the participants in conjunction with the child, when necessary, to ensure accurate reporting of behaviors such as diet, oral hygiene, and dental care access. The questionnaire covered the following domains: sociodemographic information, such as age, gender, class, and residence (urban or rural). Oral hygiene practices included frequency of tooth brushing, fluoride exposure in the form of fluoridated water, use of fluoride toothpaste, and preventive treatments (such as fluoride application or sealants). Dietary habits such as the frequency of consumption of sugary snacks, carbonated drinks, and fruit juices. Information on meal patterns and snacks consumed between meals was also recorded. The caries’ experiences of mothers, caregivers, and siblings of similar age were also recorded. The questionnaire was pilot-tested with a small group of parents/guardians and children to ensure clarity and appropriateness for the age group.

The American Academy of Pediatric Dentistry (AAPD) Caries-Risk Assessment Tool (CAT) [7] was used in our study. A detailed dental examination was conducted by trained and calibrated examiners to assess the presence of dental caries, missing teeth due to caries, presence of visible plaque, unusual tooth morphology, presence of one or more interproximal restorations, exposed root surfaces, overhanging restorations, severe dry mouth, and presence of any dental appliance [7].

Calibration of examiners

To ensure consistency in the dental examinations, all examiners performed training and calibration exercises before the start of the study. Intra-examiner and inter-examiner reliabilities were assessed through repeated examinations of a subset of participants. Kappa statistics were used to measure the level of agreement, which was found to be 89%, indicating good reliability.

Caries risk assessment

The caries risk for each participant was assessed using a combination of clinical findings and questionnaire responses. A risk classification system was developed based on CAT scores as low, moderate, and high risk. The risk assessment provided an exhaustive analysis of each child's propensity for future caries development, facilitating the implementation of targeted interventions.

Data management and statistical analysis

Data were entered into a secure database using double-entry methods to ensure accuracy, using the Statistical Package for Social Sciences (SPSS) (IBM SPSS Statistics for Windows, version 22.0, released 2013, IBM Corp., Armonk, NY). Descriptive statistics such as means, frequencies, and percentages were used to summarize the data. The relationship between caries risk and potential risk factors (e.g., sociodemographic factors, dietary habits, and oral hygiene practices) was examined using chi-square tests for categorical variables. Given the involvement of multiple comparisons, Bonferroni correction was applied to adjust for the increased risk of Type I errors. The level of statistical significance (α) was set at p < 0.05. In addition, caries risk assessment was performed by calculating the odds ratios (ORs) for each risk factor and preventive measure. This approach allows for a detailed evaluation of the influence of individual indicators on caries development, providing insights into potential preventive strategies.

## Results

Examination of caries susceptibility, taking into account factors such as age, sex, educational background, and community characteristics, revealed significant patterns. Among the various age cohorts, individuals aged 14-15 years constituted the largest portion of the sample, totaling 487 participants (24.35%), of which 75 (22.12%) were classified as high risk and 287 (25.20%) as low risk; however, this correlation was not statistically significant (p = 0.134). A noteworthy relationship was observed for gender (p = 0.001), with females representing 1,051 individuals (52.55%) of the overall sample, of which 185 (54.57%) were identified as high risk and 554 (48.64%) as low risk. Conversely, males exhibited a lower percentage of high-risk individuals, totaling 154 (45.43%), yet a greater proportion of low-risk individuals, amounting to 585 (51.36%).

For the educational attainment, 422 (21.10%) individuals in Class 4 were observed to have considerable representation within the low-risk category, whereas 74 (21.83%) participants from Class 8 exhibited the highest percentage within the high-risk group, and the overall correlation was statistically significant (p = 0.001). In relation to community demographics, 946 (47.30%) were identified as Hindus, and 112 (56.98%) were categorized as low risk. By contrast, 180 (53.10%) Muslims represented the highest proportion within the high-risk category, with a noteworthy p-value indicating a significant association between community affiliation and susceptibility to caries. These results underscored marked differences in caries risk as influenced by sex, educational level, and community, as detailed in Table 1.

**Table 1:** Demographic details of study participants and analysis of association with the chi-square test. *p < 0.05: significant. Data are presented in the form of n (%).

Variables	Categories	Total		High risk		Medium risk		Low risk		Chi-square test (p-value)
		N	%	n	5	n	%	n	%	
Age group (years)	6 and 7	334	16.70%	56	16.52%	78	14.94%	200	17.56%	0.134
	8 and 9	445	22.25%	78	23.01%	105	20.11%	262	23.00%	
	10 and 11	356	17.80%	58	17.11%	96	18.39%	202	17.73%	
	12 and 13	378	18.90%	72	21.24%	118	22.61%	188	16.51%	
	14 and 15	487	24.35%	75	22.12%	125	23.95%	287	25.20%	
Gender	Male	949	47.45%	154	45.43%	210	40.23%	585	51.36%	0.001*
	Female	1051	52.55%	185	54.57%	312	59.77%	554	48.64%	
Education	Class 3	245	12.25%	48	14.16%	65	12.45%	132	11.59%	0.001*
	Class 4	422	21.10%	65	19.17%	93	17.82%	264	23.18%	
	Class 5	398	19.90%	42	12.39%	82	15.71%	274	24.06%	
	Class 6	267	13.35%	58	17.11%	98	18.77%	111	9.75%	
	Class 7	356	17.80%	52	15.34%	102	19.54%	202	17.73%	
	Class 8	312	15.60%	74	21.83%	82	15.71%	156	13.70%	
Community	Hindu	946	47.30%	112	33.04%	185	35.44%	649	56.98%	0.001*
	Muslim	670	33.50%	180	53.10%	250	47.89%	240	21.07%	
	Sikh	227	11.35%	25	7.37%	52	9.96%	150	13.17%	
	Christian	157	7.85%	22	6.49%	35	6.70%	100	8.78%	

A total of 344 (17.20%) participants who engaged in bi-daily tooth brushing exhibited a diminished high-risk percentage relative to 1656 (82.80%) participants who brushed once per day (p = 0.026). Cavitation was observed in 1,310 (65.50%) subjects, with a notably elevated proportion of high-risk participants (p = 0.001). Individuals with routine dental check-ups demonstrated a greater prevalence of high-risk individuals; conversely, those who abstained from dental visits displayed a lower risk (p = 0.001). In terms of sugar intake, 766 (38.30%) participants acknowledged frequent consumption, with 275 (52.68%) classified as medium risk and 128 (37.76%) as high risk (p = 0.001). A substantial correlation was identified between low salivary flow and a high risk (p = 0.001). Finally, the absence of teeth was noted in 812 (40.60%) participants, revealing a significant relationship between high risk and missing teeth (p = 0.012). These results underscore the significance of dental visits, cavitation, and low salivary flow as critical determinants of the risk of caries (Table 2).

**Table 2:** Clinical parameters and evaluation of the study participants with the chi-square test. *p < 0.05: significant. Data are presented in form of n (%).

Variables	Categories	Total		High risk		Medium risk		Low risk		Chi-square test (p-value)
		N	%	n	5	n	%	n	%	
Brushing	Twice	344	17.20%	59	17.40%	70	13.41%	215	18.88%	0.026*
	Once	1656	82.80%	280	82.60%	452	86.59%	924	81.12%	
Cavitation	Present	1310	65.50%	294	86.73%	324	62.07%	692	60.76%	0.001*
	Absent	690	34.50%	45	13.27%	198	37.93%	447	39.24%	
Dental visit	Yes	730	36.50%	275	81.12%	198	37.93%	257	22.56%	0.001*
	No	1270	63.50%	64	18.88%	324	62.07%	882	77.44%	
Sugar consumption	Yes	766	38.30%	128	37.76%	275	52.68%	363	31.87%	0.001*
	No	1234	61.70%	211	62.24%	247	47.32%	776	68.13%	
Salivary flow	Yes	185	9.25%	156	46.02%	22	4.21%	7	0.61%	0.001*
	No	1815	90.75%	183	53.98%	500	95.79%	1132	99.39%	
Missing teeth	Yes	812	40.60%	135	39.82%	240	45.98%	437	38.37%	0.012*
	No	1188	59.40%	204	60.18%	282	54.02%	702	61.63%	

The investigation revealed notable correlations between diverse indicators and the occurrence of cavitation. A total of 63 individuals (26.40%) had a history of restorations, whereas 465 (35.50%) exhibited cavitation (p = 0.001, OR = 0.18). The presence of visible plaque demonstrated a robust association with cavitation, as 1,310 participants (65.50%) had visible plaque, with 745 (56.87%) displaying cavitation (p = 0.001, OR = 3.42). The diminished salivary flow was observed in 185 participants (9.25%), of which 102 (7.79%) manifested cavitation (p = 0.001, OR = 1.62). The use of orthodontic devices also revealed a significant association, with 288 participants (14.40%) utilizing these appliances and 145 (11.07%) exhibiting cavitation (p = 0.001, OR = 2.10). The presence of pits and fissures was recorded in 734 individuals (36.70%), correlating with an elevated cavitation rate (p = 0.001, OR = 0.30). Furthermore, interproximal caries were significantly associated with cavitation (p = 0.001, OR = 0.38). Participants characterized by white spots were more predisposed to cavitation (39.85%, p = 0.001, OR = 0.29). Preventative factors, such as the use of fluoridated water, fluoridated toothpaste, and mouthwash, significantly mitigated the risk of cavitation, with ORs of 10.25, 17.42, and 7.39, respectively (p = 0.001). Those who engaged in brushing their teeth twice daily demonstrated a lower incidence of cavitation than individuals who brushed less frequently (95.73%, OR = 16.04, p = 0.001). These outcomes underscore the critical role of oral hygiene practices and preventive strategies in reducing the risk of cavitation (Table 3).

**Table 3:** Risk assessment of caries considering indicators and preventive factors by the odds ratio. *p < 0.003: significant (with Bonferroni correction)

Indicators of Caries	Category	Total		Cavitation				Odd ratio	Chi-square test (p-value)
				No (n)	%	Yes (n)	%		
Previous restoration	Yes	528	26.40%	63	9.13%	465	35.50%	0.18259	0.001*
	No	1472	73.60%	627	90.87%	845	64.50%		
Visible plaque	Yes	1310	65.50%	565	81.88%	745	56.87%	3.42792	0.001*
	No	690	34.50%	125	18.12%	565	43.13%		
Salivary flow	Yes	185	9.25%	83	12.03%	102	7.79%	1.61941	0.001*
	No	1815	90.75%	607	87.97%	1208	92.21%		
Orthodontic appliances	Yes	288	14.40%	143	20.72%	145	11.07%	2.10042	0.001*
	No	1712	85.60%	547	79.28%	1165	88.93%		
Pit and fissures	Yes	734	36.70%	139	20.14%	595	45.42%	0.30315	0.001*
	No	1266	63.30%	551	79.86%	715	54.58%		
Interproximal caries	Yes	845	42.25%	191	27.68%	654	49.92%	0.38394	0.001*
	No	1155	57.75%	499	72.32%	656	50.08%		
White spots	Yes	634	31.70%	112	16.23%	522	39.85%	0.29251	0.001*
	No	1366	68.30%	578	83.77%	788	60.15%		
Caries in sibling	Yes	578	28.90%	232	33.62%	346	26.41%	1.41131	0.001*
	No	1422	71.10%	458	66.38%	964	73.59%		
Snacking	Yes	766	38.30%	254	36.81%	512	39.08%	0.90799	0.323
	No	1234	61.70%	436	63.19%	798	60.92%		
Exposed roots	Yes	865	43.25%	155	22.46%	710	54.20%	0.24483	0.001*
	No	1135	56.75%	535	77.54%	600	45.80%		
Preventive factors									
Fluoridated water	Yes	343	17.15%	267	38.70%	76	5.80%	10.2488	0.001*
	No	1657	82.85%	423	61.30%	1234	94.20%		
Fluoridated paste	Yes	453	22.65%	371	53.77%	82	6.26%	17.4168	0.001*
	No	1547	77.35%	319	46.23%	1228	93.74%		
Mouth wash	Yes	234	11.70%	176	25.51%	58	4.43%	7.39139	0.001*
	No	1766	88.30%	514	74.49%	1252	95.57%		
Brushing (two times)	Yes	344	17.20%	288	41.74%	56	4.27%	16.0426	0.001*
	No	1656	82.80%	402	58.26%	1254	95.73%		
Xylitol gum	Yes	128	6.40%	94	13.62%	34	2.60%	5.91907	0.001*
	No	1872	93.60%	596	86.38%	1276	97.40%		

## Discussion

The assessment of caries vulnerability undertaken in this study provides significant insights into the multifaceted nature of dental caries risk, influenced by an array of demographic, behavioral, and physiological determinants. These findings illuminate the intricate interrelations among individual attributes, such as age, gender, and educational attainment, alongside communal factors and personal health practices. The results align with the pre-existing body of literature concerning the etiologies of caries while also introducing novel aspects that underscore the importance of addressing both personal and environmental factors in oral health. A comprehensive understanding of these components can guide the formulation of more focused and efficacious public health strategies to alleviate the overall prevalence of dental caries.

One notable observation in this study is that adolescents aged 14-15 years represented the largest proportion of participants, accounting for 24.35% of the total sample. This finding was in agreement with a study by Bansal et al. [8], who reported a dental caries prevalence of 22.9% in the 14-to-15-year age group. Adolescence is a key period in dental health and is marked by lifestyle changes, increased independence, and shifts in oral hygiene practices. However, although this age group was well represented, the relationship between age and caries risk did not reach statistical significance (p = 0.134). This lack of significance suggests that while caries may be common among adolescents, age alone does not fully explain this risk [9]. The results indicate that behavioral factors, such as diet and oral hygiene habits, likely play a more central role in determining susceptibility to caries, even during this critical developmental phase. Therefore, public health interventions targeting caries prevention should focus not only on age-specific approaches but also on modifying behaviors and creating supportive environments for better oral health outcomes [10].

Gender disparities in vulnerability to caries were notably evident, as females exhibited a markedly greater percentage of individuals categorized as high risk (54.57%) than their male counterparts (45.43%). This gender-based difference reached statistical significance, thereby underscoring the notion that sex is a crucial factor in susceptibility to caries. These findings were consistent with those of Kumar et al. [11]. A multitude of factors may explain the elevated incidence of caries in females. Biological distinctions, including hormonal variations, may influence alterations in salivary flow and composition, rendering women more susceptible to dental complications such as caries. Societal determinants, including dietary practices and accessibility to dental services, may also be implicated [12]. Premature eruption of teeth in females may further exacerbate this issue [11]. These observations highlight the critical need to consider sex-specific variables in oral health education and care to ensure that women obtain the requisite preventive measures and guidance to alleviate their heightened vulnerability to caries.

The significance of educational attainment with caries risk emerged as another critical outcome of this investigation. Subjects hailing from higher socioeconomic strata, notably those in classes 7 and 8, exhibited a markedly elevated proportion of individuals designated as high risk. A comparable observation was documented in the research conducted by Folayan et al. [13]. This phenomenon may be attributable to their demanding schedules, filled with substantial homework from academic institutions and supplementary tutoring, which monopolizes a considerable portion of their time. These results imply that enhancing health literacy and facilitating access to dental education for less-educated demographics could substantially mitigate the incidence of caries.

The attributes of the community were also instrumental in influencing susceptibility to caries. The investigation demonstrated notable disparities in caries risk correlated with community association, where individuals identified as Muslims exhibited the highest percentage of high-risk cases, whereas Hindus were more prominent within the low-risk group. A comparable observation was documented in a study by Al-Meedani et al. [14]. This association was statistically significant, suggesting that cultural, religious, and socioeconomic factors within communities may influence oral health behaviors [15]. Moreover, the accessibility of healthcare provisions, which include dental services, is subject to variation based on community assets and infrastructural capabilities. These insights highlight the importance of considering community-specific factors in oral health programs, as certain demographic groups may require more tailored approaches to address their unique risk determinants [16].

Oral hygiene practices demonstrated a strong correlation with the risk of caries, as evidenced by participants who engaged in bi-daily tooth brushing, exhibiting a markedly lower percentage of high-risk individuals than those who brushed only once each day. Congruent findings have been documented in previous research [13,16,17]. This observation is consistent with existing literature that underscores the essential function of frequent and effective tooth brushing in mitigating plaque accumulation and diminishing the likelihood of caries development. In addition, regular dental examinations were associated with improved identification of high-risk individuals, whereas those who neglected dental appointments were more likely to remain undiagnosed. This research further identified a significant correlation between sugar intake and heightened caries susceptibility, thereby reaffirming the established relationship between elevated sugar consumption and the onset of dental caries. These findings underscore the necessity for public health initiatives that advocate for consistent oral hygiene practices, regular dental examinations, and decreased sugar consumption to mitigate the risk of caries [16].

Physiological variables such as the rate of salivary secretion and cavitation have been identified as crucial factors influencing caries' vulnerability. A notable correlation exists between diminished salivary flow and elevated risk of caries. Saliva performs various protective functions, including dilution and cleansing of the oral cavity, provision of host defense mechanisms, as well as buffering, and facilitating ion exchange; deviations in specific salivary characteristics from standard levels may contribute to caries pathogenesis [18]. A reduction in salivary flow compromises the inherent defense of the mouth against caries, thereby increasing the probability of dental decay. Comparable conclusions were drawn by Munoz et al. [19], who found a significant association between the occurrence of at least one decayed tooth and salivary flow rate. The investigation further emphasized the significance of cavitation, as 65.50% of the subjects displayed discernible plaque, which is a fundamental factor in the etiology of dental caries. The correlation between plaque presence and cavitation is notably significant, highlighting the critical necessity for efficient plaque eradication in the prevention of carious lesions [20].

Preventive strategies, including the use of fluoridated water, toothpaste, and mouthwash, have been demonstrated to be significantly effective in reducing the likelihood of cavitation [21]. Fluoride enhances the strength of tooth enamel and contributes to the mitigation of decay. The findings of this research endorse its ongoing application as an essential element in caries prevention methodologies. In addition, participants who engaged in bi-daily tooth brushing exhibited a reduced frequency of cavitation, thereby further emphasizing the critical role of consistent and appropriate oral hygiene practices in dental health preservation.

Clinical implications

The results of this investigation hold considerable significance in the formulation of public health policies and intervention frameworks. The evident correlations among demographic variables, such as sex, educational background, and community attributes, in conjunction with oral hygiene practices and physiological markers, indicate that initiatives aimed at caries prevention must be diverse and customized to meet the unique requirements of various demographic groups. Public health initiatives should concentrate on enhancing health literacy, especially among those with lower levels of education, while also advocating gender-sensitive methodologies in oral healthcare. Furthermore, strategies to diminish sugar intake, augment accessibility to fluoride products, and promote routine dental check-ups should be prioritized as integral components of a holistic approach to mitigating the prevalence of dental caries.

Limitations

This study is cross-sectional in nature, so it can only establish associations and not causality. In addition, self-reported data on dietary habits and oral hygiene practices may be subject to recall or social desirability bias. However, efforts will be made to minimize these limitations by providing clear instructions and verifying the responses during the interviews.

## Conclusions

The findings of this investigation revealed notable correlations among demographic variables, oral sanitation behaviors, and physiological states with susceptibility to caries and cavitation. Sex, level of education, and community ties were significant determinants of caries risk, whereas consistent tooth brushing, regular dental check-ups, and the utilization of fluoridated products proved to be effective preventive strategies. Diminished salivary flow, the presence of visible plaques, and the use of orthodontic devices markedly increased the risk of cavitation. These results emphasize the necessity for focused public health initiatives, the advancement of oral hygiene education, and the enhancement of access to dental services to mitigate the prevalence of caries and foster overall oral health.
